# The Posttraumatic Increase of the Adhesion GPCR EMR2/*ADGRE2* on Circulating Neutrophils Is Not Related to Injury Severity

**DOI:** 10.3390/cells12222657

**Published:** 2023-11-20

**Authors:** Leyu Zheng, Moujie Rang, Carolin Fuchs, Annette Keß, Mandy Wunsch, Julia Hentschel, Cheng-Chih Hsiao, Christian Kleber, Georg Osterhoff, Gabriela Aust

**Affiliations:** 1 Research Laboratories and Department of Orthopaedics, Trauma and Plastic Surgery (OUP), Leipzig University and University Hospital Leipzig, 04103 Leipzig, Germany; leyu.zheng@medizin.uni-leipzig.de (L.Z.); moujie.rang@medizin.uni-leipzig.de (M.R.); carolin.fuchs@medizin.uni-leipzig.de (C.F.); annette.kess@medizin.uni-leipzig.de (A.K.); mandy.wunsch@medizin.uni-leipzig.de (M.W.); christian.kleber@medizin.uni-leipzig.de (C.K.); georg.osterhoff@medizin.uni-leipzig.de (G.O.); 2 Institute of Human Genetics, Leipzig University and University Hospital Leipzig, 04103 Leipzig, Germany; julia.hentschel@medizin.uni-leipzig.de; 3 Department of Experimental Immunology, Amsterdam Institute for Infection and Immunity, Amsterdam University Medical Centers, 1105 AZ Amsterdam, The Netherlands; c.hsiao@amsterdamumc.nl; 4 Research Laboratories and Department of Visceral, Transplantation, Vascular and Thoracic Surgery (VTTG), Leipzig University and University Hospital Leipzig, 04103 Leipzig, Germany

**Keywords:** EMR2, adhesion G-protein coupled receptor, neutrophils, polytrauma, DAMP, severe injured patient

## Abstract

Trauma triggers a rapid innate immune response to aid the clearance of damaged/necrotic cells and their released damage-associated molecular pattern (DAMP). Here, we monitored the expression of EMR2/*ADGRE2*, involved in the functional regulation of innate immune cells, on circulating neutrophils in very severely and moderately/severely injured patients up to 240 h after trauma. Notably, neutrophilic EMR2 showed a uniform, injury severity- and type of injury-independent posttraumatic course in all patients. The percentage of EMR2^+^ neutrophils and their EMR2 level increased and peaked 48 h after trauma. Afterwards, they declined and normalized in some, but not all, patients. Circulating EMR2^+^ compared to EMR2^−^ neutrophils express less CD62L and more CD11c, a sign of activation. Neutrophilic EMR2 regulation was verified in vitro. Remarkably, it increased, depending on extracellular calcium, in controls as well. Cytokines, enhanced in patients immediately after trauma, and sera of patients did not further affect this neutrophilic EMR2 increase, whereas apoptosis induction disrupted it. Likely the damaged/necrotic cells/DAMPs, unavoidable during neutrophil culture, stimulate the neutrophilic EMR2 increase. In summary, the rapidly increased absolute number of neutrophils, especially present in very severely injured patients, together with upregulated neutrophilic EMR2, may expand our in vivo capacity to react to and finally clear damaged/necrotic cells/DAMPs after trauma.

## 1. Introduction

Injuries, both unintentional and violence-related, take the lives of five million people worldwide each year and constitute 10% of all deaths (https://www.who.int; accessed on 19 August 2023). Tens of millions more people suffer non-fatal injuries each year. Polytrauma results in an imbalanced pro- and anti-inflammatory/resolving immune response leading to immune dysfunction [1,2]. It is triggered by the tremendous release of damage-associated patterns (DAMPs) from damaged, dying or dead cells and inflammatory mediators such as complement and cytokines [2,3,4]. Innate immune cells, especially neutrophils, become activated and assume the first line of immune defense against DAMPs (reviewed in [5]).

Adhesion/class B2 G protein-coupled receptors (aGPCRs) are a structurally separate class of GPCRs [6,7,8]. They have an extended N-terminal extracellular domain (ECD) that contains various protein folds, facilitating cellular adhesion to and interaction with other cell surface receptors or extracellular matrix constituents, and the GPCR autoproteolysis inducing domain (GAIN), covering its internal GPCR proteolysis site (GPS). The receptors are self-cleaved at the GPS into two non-covalently associated fragments (reviewed in [9]).

Of the 33 aGPCR members in humans, 8 are present on hematopoietic stem, progenitor and/or mature cells [10]. All family ‘E’ members, compromising EMR1/*ADGRE1*, EMR2/*ADGRE2*, EMR3/*ADGRE3* and CD97/*ADGRE5* in humans, are expressed in innate immune cells. They possess N-terminal repetitive, alternatively-spliced epidermal growth factor (EGF)-like adhesive folds. Their number and sequence determine the adhesion of these receptors to several binding partners [11].

The expression of EMR2 on myeloid cells such as granulocytes and monocytes/macrophages [10,12,13] suggests that this aGPCR is involved in the regulation of the innate immune response. Indeed, the activation of EMR2 by monoclonal antibody (mAb) ligation regulates neutrophil responses by potentiating the effects of pro-inflammatory mediators [14], and changes the cytokine secretion profile and survival of lipopolysaccharide (LPS)-treated neutrophils [15]. In blood-derived monocytes, the EMR2 mAb ligation or EMR2 binding of a complex consisting of factor H-related protein 1 (FHR1)/necrotic-type cells triggers the phospholipase C (PLC)-pathway and the assembly of the NLPR3 inflammasome [16,17]. This signaling cascade finally leads to caspase 1-dependent release of pro-inflammatory cytokines IL-1β and IL-18. NLPR3 represents the principal inflammasome activated in innate immune cells through DAMP recognition, finally leading to pyroptosis [18].

In contrast to the well-clarified signaling cascades following EMR2 activation, there are very little data on what regulates EMR2 expression in innate immune cells. Under which (patho)physiological conditions EMR2 is up- or downregulated and what the potential stimuli or suppressors for EMR2 expression are remains unclear. Clinical data indicate that EMR2 increases in circulating neutrophils in patients with non-infectious systemic inflammatory response syndrome (SIRS) compared to healthy controls [14,19], but neutrophilic EMR2 had been quantified only once in these patients.

Here, we examined the posttraumatic course of EMR2 expression in circulating neutrophils in traumatized patients with a wide range of injury severity scores (ISSs) to calculate the effects of the traumatic event on EMR2. The data, obtained at six defined time points up to 10 days after trauma, were related to time-matched clinico-pathological parameters of the patients. Finally, we verified in vitro the stimuli/conditions upregulating neutrophilic EMR2 after trauma.

## 2. Materials and Methods

### 2.1. Ethics Statement and Clinical Study of Traumatized Patients

The prospective study was approved by the local ethics committee at the Leipzig University (reference number 188-17lk). Samples and data were collected with informed written consent of patients or their legal representatives and of uninjured volunteers. Patients were excluded if one or more of the following criteria were fulfilled: age < 18 years, time between trauma and hospital admission > 1 h, life expectancy < 24 h, participation in other trials, cardiopulmonary reanimation at scene of the accident, dying immediately after hospital admission, known or suspected pregnancy, and radio- or chemotherapy within the last 3 months. We included 34 trauma patients; their median age was 54.0 (34.0–71.5) years and 73.5% were male. The ISS was estimated after whole-body computed tomography and was confirmed retrospectively after hospital discharge. It varied from 9 to 66 (33.5, 21.3–41.5), that is, from moderate to very severely injured. By definition, an ISS of 1–8 is considered minor, 9–15 moderate, 16–24 severe, and 25 and higher very severe [20]. The patients were divided into two groups: ISS < 25 (9–24, *n* = 9) moderately/severely injured and ISS ≥ 25 (*n* = 25) very severely injured; patients’ main characteristics are shown in Table 1; for information concerning the type of injury see Table S1. All body regions, classified based on the abbreviated injury scale (AIS) [21], were involved in injuries.

Especially in patients with an ISS < 25, daily scores such as the Sequential Organ Failure Assessment (SOFA) score could not be generated in all cases after 24 h because patients often left the intensive care unit (ICU), and thus the parameters of such scores were not further evaluated. The study included five volunteers who underwent the same time course in blood taking as the patients but experienced no injury and were not hospitalized. The median volunteer group age was 51.0 (41.0–60.0) years, and three of the participants were male. Blood was taken once from a further 16 uninjured volunteers for in vitro experiments.

### 2.2. Blood Sample Preparation and Determination of Parameters

Blood was obtained at 1, 8, 24, 48, 120, and 240 h upon admission to the hospital. Tolerance for blood taking was ±10%/time span. Leukocytes were prepared within 15 min after blood draw. 2.5 mL EDTA-treated blood was added to 22.5 mL Schwinzer red blood cell lysis solution [22] and incubated for 10 min at 4 °C. The cells were washed twice with PBS (pH 7.4) at 300× *g*. For patients, the full leukocyte cell count was determined in EDTA-blood in an automatic blood cell analyzer (XN-9000 Sysmex GmbH, Norderstedt, Germany) at each time point. To obtain serum or EDTA-plasma, the respective Monovette (Sarstedt AG, Nümbrecht, Germany) was centrifuged at 2000× *g* for 10 min at 20 °C. The supernatants were transferred into encoded cryotubes, stored at −80 °C, and thawed only once for analysis. IL-6, IL-8, and CCL2 (MCP-1) were quantified in sera using the human IL-6, IL-8, and MCP-1 BD OptEIA ELISA kits (Becton Dickinson GmbH, Heidelberg, Germany). C-reactive protein (CRP) was quantified in sera by immunoturbidimetry and procalcitonin (PCT) in plasma using TRACE technology (Roche Deutschland Holding GmbH, Grenzach-Wyhlen, Germany).

### 2.3. CFHR1 Genotype Assessment

The copy number variation (CNV) analysis of *CFHR1*/*CFHR3* was carried out using multiplex ligation-dependent probe amplification (MLPA) with the P236-B1 probe mix, according to manufacturer’s instructions (MRC Holland, Amsterdam, The Netherlands). Fragment analysis was carried out on a Genetic Analyzer ABI3500 (Thermo Fisher Scientific, Darmstadt, Germany) and Gene Mapper Software 5.0. Data analysis was performed using the MLPA module of JSI SequencePilot (JSI Medical Systems, Ettenheim, Germany).

### 2.4. Flow Cytometric Analysis of Patients’ Leukocytes

Each Ab (Table S2) was titrated for optimal concentration to establish a multi-color Ab-panel, not shown completely, for circulating myeloid cells. To prevent unspecific Ab binding, the Fc-blocking agent (Miltenyi Biotec GmbH, Bergisch Gladbach, Germany) was added 10 min before Ab staining. Two leukocyte samples were prepared at each time point. The first contained all Abs, and the second was a fluorescence minus one (FMO) control, in which the interesting Abs were replaced by isotype-specific controls labeled with the respective fluorophores. The Ab-panel was applied for 30 min at 4 °C, the cells were washed three times and analyzed immediately using flow cytometry. Before each measurement, the flow cytometer (BD LSRFortessa^TM^ X-20 Cell Analyzer; Becton Dickinson) was calibrated using Sphero™ Rainbow Calibration Particles (Becton Dickinson). The datasets of all individuals were analyzed using FlowJo Software 10 (FlowJo LLC, Ashland, OR, USA). For quality control of the fluorescence measurements over a long time, the FlowClean plugin was applied.

**Figure 1:**
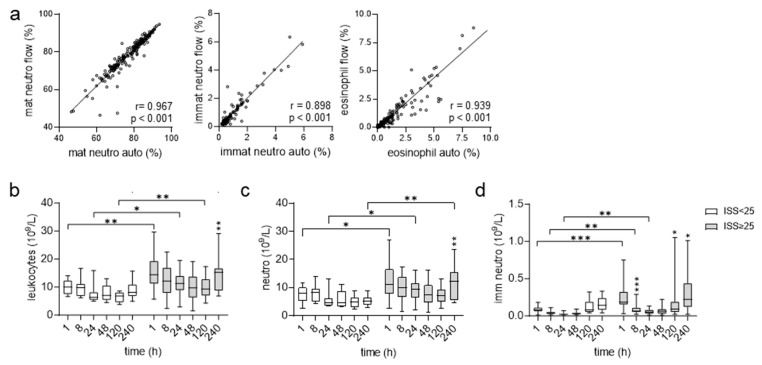
Posttraumatic course of leukocyte blood counts.(**a**) Correlation of the percentage of subsets within all leukocytes (set 100%) quantified via flow cytometry (flow) or an automatic blood cell analyzer (auto); *n* = 32 patients, *n*= 186 time points; Spearman’s correlation coefficient r and *p* values are shown. (**b**–**d**) Absolute numbers of circulating leukocytes (**b**), mature (**c**), and immature neutrophils (**d**) in patients 1–240 h after trauma, determined using an automatic analyzer. Comparison between patient groups ISS < 25 (9–24) and ISS ≥ 25, *t*-test (leukocytes, mature neutrophils), U-test (immature neutrophils); comparison between consecutive time points in one patient group: ANOVA; only significant changes related to the previous time point are shown; * *p* < 0.05, ** *p* < 0.01, *** *p* < 0.001.

The main gating and analysis strategy for neutrophils is shown in Figure S1a,b. Granulocytes were separated from monocytes, dendritic, natural killer (NK), and T/B-cells via CD14, CD56, HLA-DR, and CD3/CD19/CD20 Ab staining. Using CD16/CD45, we separated neutrophils from eosinophils, which are CD16^−^ CD45^high^. Mature granulocytes are CD16^high^ CD45^mid^. The CD16^low^ CD45^mid^ subset contains immature granulocytes [23,24]. Both neutrophil subsets were analyzed for the percentage of EMR2^+^ cells, and the median fluorescence intensity (MFI) of EMR2 expression (Figure S1b). To verify the quality of our flow cytometric analyses, we compared the percentage of leukocyte subsets among all leukocytes quantified either via flow cytometry or the automatic blood cell analyzer. The percentages of neutrophils and eosinophils correlated strongly between both methods (Figure 1a). Thus, the relative percentage of EMR2^+^ cells can be related to absolute cell numbers.

### 2.5. Leukocytes and Neutrophils for In Vitro Experiments

To investigate in vitro the conditions/stimuli upregulating neutrophilic EMR2 in trauma patients, we initially compared methods for leukocyte isolation and neutrophil enrichment. Cell purity and viability were quantified through side- (SSC-A) and forward (FSC-A) scatter analyses and staining with the Fixable Viability Dye BV605 (Becton Dickinson) in flow cytometry. The loss of CD16 in isolated or enriched cells was quantified as an indicator for programmed cell death induction in neutrophils [25]. A 1 g sedimentation over 50 min and two washing steps at 20 g for 20 min [26] preserved viability and CD16^high^ surface expression over 17 h in vitro as in leukocytes isolated via red blood cell lysis, but this method took more time. To enrich neutrophils, density gradient centrifugation at 550 g/30 min using Polymorphprep was used (Serumwerk Bernburg, Germany), but yielded insufficient purity. A multistep protocol including the removal of platelets and a two-time density gradient of histopaque 1119 and 1077 (Sigma-Aldrich GmbH, Taufkirchen, Germany) at 700 g/45 min centrifugation [27] avoided the release of cell debris via red blood cells lysis. However, damaged/dying cells and cell debris were still present, as seen through unbiased scatter analysis in flow cytometry; thus, neutrophils were truly enriched only up to ~70%. Partly, these cells lost CD16 early in vitro. Overall, high g-forces and centrifugation times have a paralytic impact on neutrophils [26]. Finally, we decided to use leukocytes after red blood cell lysis.

### 2.6. In Vitro Experiments

To verify whether a patient’s circulating EMR^2−^ and EMR2^+^ neutrophils differ in their activation status, isolated leukocytes were either left untreated or stimulated with 10 ng/mL TNFα (ImmunoTools GmbH, Friesoythe, Germany) for 20 min, stained for CD45, EMR2, CD62L, CD11b, and CD11c and analyzed using flow cytometry.

To investigate EMR2 upregulation in vitro, the wells for leukocyte incubation (37 °C, 5% CO_2_) were either pre-coated with 5 μg/mL FHR1 (Sigma-Aldrich) or bovine serum albumin (BSA) for 1 h. Each 5 × 10^5^ leukocytes of uninjured volunteers were incubated for 0.5 up to 17 h either in RPMI1640/10% FCS (control) or in 10% human serum or plasma taken from patients 24 h after trauma or from uninjured volunteers. Amounts of 1 mM EDTA and 10 μM BAPTA-AM (Cayman Chemical, Ann Arbor, MI, USA) were added to chelate calcium. Amounts of 2 ng/mL rh IL-1b, 10 ng/mL rh IL-6, 2 ng/mL rh IL-8, 2 ng/mL rh IL-17b, 10 ng/mL rh CCL2 (MCP1), 10 ng/mL rh TNFa (all Immunotools), or 1 mM LPS (Sigma-Aldrich) were added to verify the role of cytokines and LPS, respectively. The impact of intrinsic and extrinsic apoptosis was quantified with 1 μM staurosporin (Hycultec GmbH, Beutelsbach, Germany) and 5 ng/mL TNFα/10 µg/mL cycloheximide (Biozol GmbH, Eching, Germany), respectively. A total of 20 mM Z-VAD-FMK (ENZO Life Sciences, Lörrach, Germany) was used to examine the role of caspases induced during apoptosis. Necrotic leukocytes were generated via three-time repeated freeze/thaw cycle of 2 min liquid nitrogen and 4 min 37 °C or via heat (65 °C, 5–45 min) [16,28]. The 5 × 10^5^ necrotic cells and their released components were added to 5 × 10^5^ leukocytes. Force was applied via orbital shaking (300 rpm). The cells were stained first with the Viability Dye, afterwards blocked for Fc-receptor binding, stained for EMR2 and other cell surface receptors and analyzed via flow cytometry. 

### 2.7. ADGRE2 qRT-PCR Analysis

The 4 × 10^6^ leukocytes were homogenized in 300 µL RLT Buffer (Qiagen, Hilden, Germany) and total RNA was isolated according to the manufacturer’s instructions. For cDNA synthesis, 0.1 μg RNA was transcribed using the SuperScript™ IV First-Strand Synthesis System (Thermo Fisher Scientific). The relative levels of cDNA were quantified using a Rotor-Gene RG3000 (Qiagen, Hilden, Germany) with the ΔΔCt method. The expression of specific genes was normalized to *RLP27*. Primer sequences can be provided upon request.

### 2.8. Statistics

Normally distributed continuous variables were presented as mean ± standard error of mean (SEM), non-normally distributed as median and the interquartile range (25th–75th percentile). Data were analyzed using SPSSv27 (IBM, Armonk, NY, USA) and GraphPad Prism10 (GarphPad Software, Boston, MA, USA). *p* values were two-sided and α < 0.05 was used for hypothesis testing.

## 3. Results

### 3.1. Higher Absolute Blood Counts for Neutrophils in Very Severely Injured Patients

First, we compared moderately/severely with very severely injured patients for basic diagnostic data. The leukocyte count was higher in very severely injured patients at most posttraumatic time points (Figure 1b), confirming that leukocytosis after trauma is related to injury severity [29]. Consistent with this is the higher neutrophil count at many time points in these patients (Figure 1c), underscoring that neutrophilia is one of the first changes following trauma [5]. The typical early posttraumatic increase in the immature neutrophil count [5] was seen especially in the very severely injured patients (Figure 1d). In total, we confirmed well-established clinical data and, importantly, both patient groups differ in the absolute number of (immature) neutrophils.

### 3.2. Uniform Posttraumatic Course of EMR2 Expression on Neutrophils in Injured Patients

**Figure 2:**
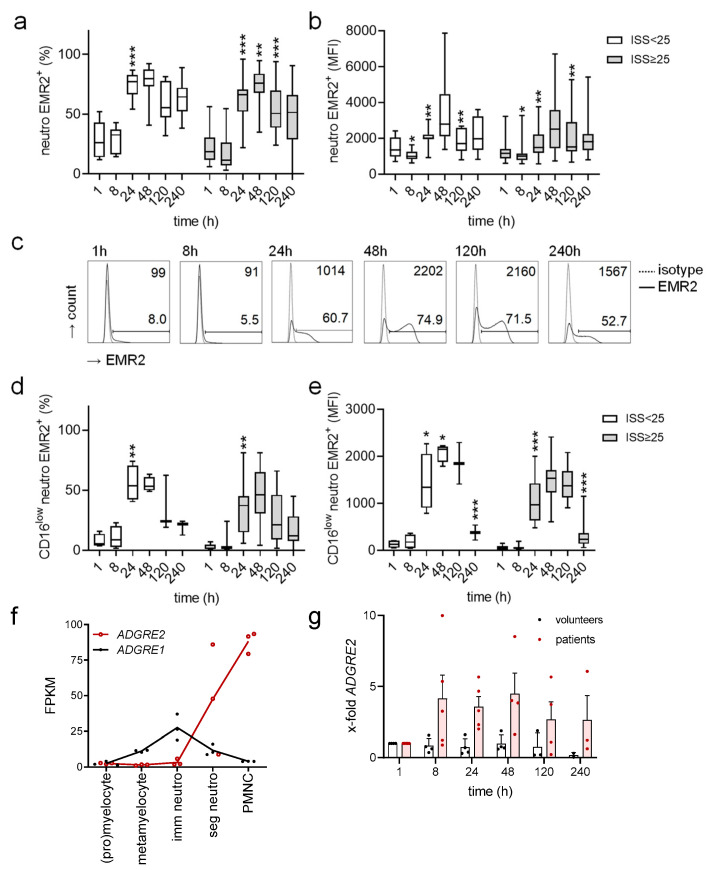
Posttraumatic course of EMR2 in circulating neutrophils. (**a**,**b**) Percentage of EMR2^+^ mature (CD16^high^) neutrophils (**a**) and median fluorescence intensity (MFI) of EMR2 (**b**) in these cells. (**c**) Time course of EMR2 expression on neutrophils of one typical patient. The percentage of EMR2^+^ cells is indicated in the graph; the MFI of EMR2^+^ cells (at 1 and 8 h isotype control) is shown in the upper right corner. (**d**,**e**) Percentage of CD16^low^ EMR2 neutrophils (**d**) and their EMR2 MFI (**e**). (**a**–**e**) Comparison between patient groups ISS < 25 (9–24) and ISS ≥ 25: *t*-test; comparison between consecutive time points in one patient group: ANOVA, only significant changes related to the previous time point are shown; * *p* < 0.05, ** *p* < 0.01, *** *p* < 0.001. (**f**) The expression of *ADGRE2*, encoding EMR2, during the maturation of myeloid cells. RNA sequencing data were derived from [30]. *ADGRE2* is upregulated during the maturation of neutrophils. *ADGRE1*, encoding EMR1, is present only in circulating eosinophils [19]. Thus, it decreased after the immature stage. FPKM, fragments per kilobase per million mapped reads; PMNC, peripheral blood polymorphonuclear cells; imm, immature; seg, segmented neutrophils. (**g**) Quantitation of *ADGRE2* via qRT-PCR in circulating leukocytes in the posttraumatic course; *n* = 5 injured patients (1/5 patient only 1–48 h), *n* = 4 uninjured volunteers (1/4 only 1–48 h); mean ± SEM.

The percentage of mature EMR2^+^ (CD16^high^ CD45^mid^) neutrophils and EMR2 expression level in these cells showed a characteristic, uniform posttraumatic course in all injured patients (Figure 2a–c). Both parameters were low from 1 to 8 h after trauma. Afterwards, they increased, reaching the maximum at 48 h. At 120–240 h after trauma, the percentage of EMR2^+^ neutrophils and their EMR2 expression level declined but remained at a higher level 120–240 h compared to 1–8 h after trauma. Surprisingly, the posttraumatic course of EMR2 was seen in all patients and was not associated with injury severity and type of injury.

In CD16^low^ CD45^mid^ neutrophils, mainly comprising immature neutrophils, we observed a similar posttraumatic course of EMR2 expression (Figure 2d,e). However, the percentage of EMR2^+^ CD16^low^ neutrophils 24–240 h after trauma was lower and immature neutrophils expressed less EMR2 compared to mature ones (Figure 2b,e). Indeed, neutrophil maturation and differentiation are paralleled by increased *ADGRE2* levels, as reanalyzed published data [30] show (Figure 2f).

In healthy people, EMR2 expression on neutrophils is low [19,31,32]. To verify how EMR2 varies over 240 h, the same period as for injured patients, we examined uninjured, non-hospitalized volunteers (Figure S2a,b). The percentage of EMR2^+^ neutrophils varied between them but remained constant for single individuals (Figure S2c). Interestingly, several volunteers showed repeatedly increased neutrophilic EMR2 levels compared to others (Figure S2c).

In summary, EMR2 was upregulated in mature and immature neutrophils after trauma. To verify whether this is caused by protein synthesis, we quantified *ADGRE2* in the posttraumatic course (Figure 2g). Because very little blood could be taken from injured patients, leukocytes were analyzed. *ADGRE2* mRNA increased at 8 h and remained high at 120–240 h in trauma patients but not in uninjured volunteers.

### 3.3. Circulating EMR2^+^ Neutrophils Express Less CD62L and More CD11c Compared to EMR2^−^

**Figure 3:**
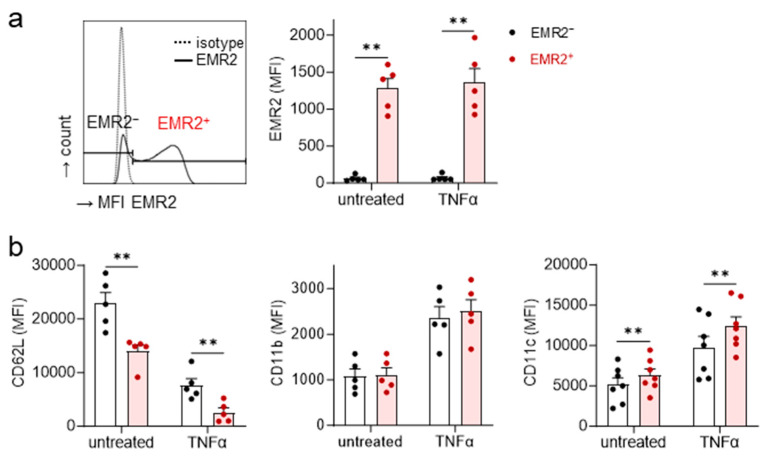
EMR2^−^ and EMR2^+^ neutrophils differ in the expression of activation-related CD62L and CD11c. Leukocytes of polytrauma patients (*n* = 6, 24 h after trauma) were left untreated or stimulated with 10 ng/mL TNFα for 20 min, mAb-stained, and analyzed via flow cytometry. (**a**) The EMR2^-^ and EMR2^+^ neutrophils were separately analyzed for (**b**) CD62L, CD11b, and CD11c; mean ± SEM, paired *t*-test, ** *p* < 0.011.

After trauma, a subset of mature (CD16^high^) and a subset of CD16^low^ neutrophils expressed EMR2. We quantified EMR2^−^ and EMR2^+^ mature neutrophils for activation-related molecules (Figure 3a,b). TNFα, a strong neutrophil activator and used as a positive control, left EMR2 unchanged but lowered CD62L and enhanced CD11b and CD11c (Figure 3b), confirming well-known data [33]. Untreated or TNFα-stimulated EMR2^+^ neutrophils expressed less CD62L and more CD11c compared to EMR2^−^ neutrophils, but CD11b was not changed (Figure 3b). Thus, EMR2^+^ compared to EMR2^−^ neutrophils were more activated or primed for activation.

Not shown in detail, the EMR2^−^ and EMR2^+^ cells in each mature and immature neutrophils neither represent the CD16^high^ CD62^low^ or the CD16^low^CD62L^high^ subset present in healthy volunteers and changed in their percentages among all neutrophils in trauma patients [23].

### 3.4. Posttraumatic Levels of EMR2 Expression on Neutrophils and C-Reactive Protein (CRP) Correlate

**Figure 4:**
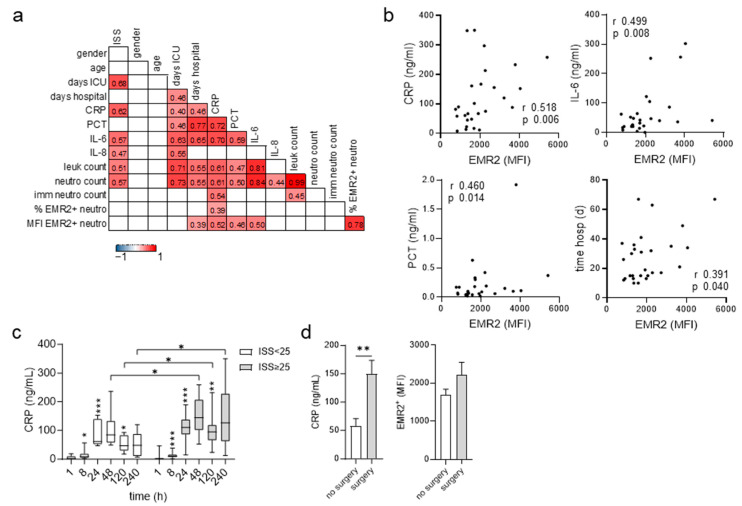
Expression of EMR2 on circulating neutrophils after trauma and its correlation to clinic. (**a**,**b**) Correlation of the percentage of EMR2^+^ neutrophils and the expression level of EMR2 on these cells to clinicopathological parameters 240 h after trauma. In the correlation matrix (**a**), square color indicates the magnitude of correlation; only significant correlations (Spearman’s) are shown. In (**b**), the significant correlations of the expression level of EMR2 on neutrophils to CRP, IL-6, PCT, and days of hospitalization are visualized. (**c**) Posttraumatic course of CRP, comparison between patient groups ISS < 25 (9–24) and ISS ≥ 25, *t*-test; comparison between consecutive time points in one patient group: ANOVA, only significant changes related to the previous time point are shown; * *p* < 0.05, ** *p* < 0.01, *** *p* < 0.001. (**d**) Circulating CRP levels and expression levels of EMR2 on neutrophils in patients 240 h after trauma. Patients who underwent surgery (*n* = 16) within 120–240 h after trauma were compared to patients not operated on in this period; unpaired *t*-test, ** *p* < 0.01).

At 240 h after trauma the EMR2 level on neutrophils was still high in many patients compared to uninjured volunteers. In correlation analyses, EMR2 was most related to CRP and IL-6 (Figure 4a,b). The posttraumatic course of measurable CRP was similar to that of EMR2 expression on neutrophils and was also seen in all patients: after a lag phase of several hours, the measurable CRP increased, peaked at 48 h, and decreased (Figure 4c). Very severely injured patients had higher CRP levels at 48–240 h after trauma (Figure 4c). Interestingly, the function of CRP entails the recognition, disposal, and clearance of dying/dead cells and their products [34]. Each surgical intervention increases CRP [35]. Indeed, patients operated on between 120–240 h after trauma showed a ~3-fold higher CRP compared to patients who did not undergo surgery and tended to also display higher neutrophilic EMR2 (Figure 4d).

### 3.5. Extracellular Calcium-Dependent Increase in EMR2 Expression on Neutrophils In Vitro

**Figure 5:**
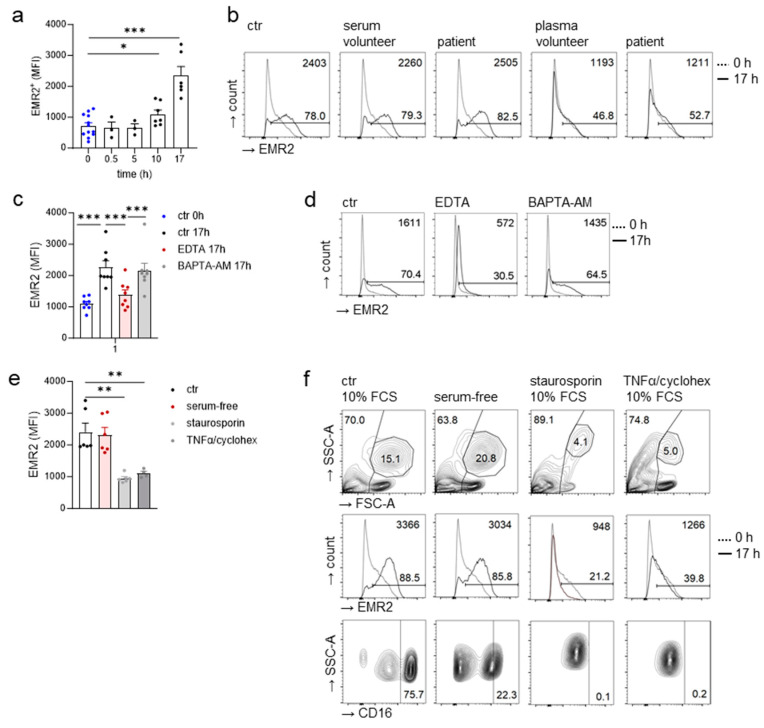
Regulation of EMR2 expression on neutrophils in vitro. (**a**–**d**) Ca^2+^-dependent increase in EMR2 expression on neutrophils during culture. Peripheral leukocytes of uninjured volunteers were cultured in medium/10% FCS (control, ctr) for the indicated time, some EDTA and BAPTA-AM was added. EMR2 was analyzed on mature neutrophils using flow cytometry. (**a**) EMR2 (MFI) on neutrophils; *n* = 3–14 volunteers/time point, * *p* < 0.05, *** *p* < 0.001, unpaired *t*-test. (**b**) Leukocytes were incubated in medium with 10% FCS (ctr), serum, or plasma of either patients or volunteers for 17 h. Patients serum and plasma were taken 24 h after injury. One typical experiment is shown. The percentage of EMR2^+^ neutrophils after 17 h is indicated in the graph, the EMR2 MFI is shown in the upper right corner. (**c**,**d**) EDTA, but not BAPTA-AM, prevented the increase in EMR2 expression on neutrophils in vitro. (**c**) Leukocytes of volunteers (*n* = 8) were incubated in medium/10% FCS (ctr) for 17 h without or with 1 mM EDTA or 1 μM BAPTA-AM; *** *p* < 0.001, paired *t*-test. (**d**) One typical experiment is shown. The percentage of EMR2^+^ neutrophils after 17 h is indicated in the graph, MFI of EMR2^+^ cells is shown in the upper right corner. (**e**,**f**) EMR2 expression on apoptotic neutrophils. Leukocytes were either cultured in serum-free medium or in medium/10% FCS (ctr) and with staurosporin or TNFα/cycloheximid for 17 h. (**e**) EMR2 MFI was quantified on neutrophils using flow cytometry; *n* = 4–6 volunteers/condition, Mann–Whitney U-test, ** *p* < 0.01. (**f**) One typical experiment is shown. Upper panel: scatter analysis of all cells (pre-gating to single cells only). The percentage of neutrophils and dead cells/cells debris (low forward scatter, FSC-A) is indicated. Middle and lower panel: the percentage of neutrophils expressing EMR2 and CD16 was quantified and indicated at/or in the gate; MFI of EMR2^+^ neutrophils is shown in the upper right corner.

To identify the stimuli upregulating EMR2 expression on patient’s neutrophils in vivo, leukocytes were investigated in vitro up to 17 h after isolation. Table 2 gives an overview of conditions/stimuli tested. First, we examined whether serum or plasma, taken from patients 24 h after trauma, contained the stimuli upregulating EMR2 expression on neutrophils in the posttraumatic course. Unexpectedly, EMR2 expression on neutrophils already increased in the control, medium with 10% fetal calf serum (FCS), with lengthening culture time (Figure 5a). A total of 10% serum from patients, but also from volunteers, increased neutrophilic EMR2 expression to the same extent (Figure 5b). A total of 10% plasma from both donor groups upregulated neutrophilic EMR2 less, indicating the role of Ca^2+^ in this process. Indeed, the addition of 1 mM EDTA, an extracellular calcium chelator, but not of 1 μM BAPTA-AM, a cell-permanent intracellular Ca^2+^ chelator (Figure 5c,d), inhibited the neutrophilic EMR2 increase. In all experiments, the previous removal of monocytes from leukocytes through adherence to a plastic surface for 60 min at 37 °C did not alter the results concerning neutrophilic EMR2, excluding monocyte-induced EMR2 regulation.

### 3.6. Inflammatory Cytokines Do Not Regulate the Neutrophilic EMR2 Increase In Vitro

We investigated further conditions that may upregulate neutrophilic EMR2 in vitro more compared to the control. These data are not shown in detail but are summarized in Table 2. Severe injury causes a systemic cytokine storm associated with adverse outcome [36,37]; thus, inflammatory cytokines are potential candidates. We found enhanced levels of IL-6, IL-8, and CCL2 (MCP1) in patients but not in uninjured volunteers. Very severely injured patients compared to moderately/severely injured ones showed higher IL-6 and IL-8 levels 8 h after trauma (Figure S3), confirming published data [36]. IL-1 and TNFα slightly increased at 1–8 h after trauma in a few patients. However, none of these cytokines, also known to stimulate hepatic CRP synthesis [38], further increased neutrophilic EMR2 in vitro when applied alone or as a cocktail. LPS, a pathogen-associated molecular pattern (PAMP) upregulating EMR2/*ADGRE2* in differentiated monocyte-derived macrophages in vitro [31], also left neutrophilic EMR2 unchanged. Finally, mechanical force, which leukocytes are exposed to in circulation, with many aGPCRs being mechanosensitive [22], and in vivo vibration activates a missense substituting EMR2 in patients with vibratoria urticaria [39], was applied via orbital shaking. It did not change neutrophilic EMR2.

### 3.7. FHR1 Is Not Involved in Neutrophilic EMR2 Upregulation In Vitro

After trauma, necrotic cells and DAMPs are released into the circulation. Interestingly, in vitro EMR2 expression on monocytes recognizes the complex consisting of necrotic-type cells and FHR1, a complement regulatory protein of the factor H protein family [40]. The binding of complexed FHR1 to EMR2 stimulates the monocytic NLPR3 inflammasome [16]. Thus, we investigated whether FHR1 and/or necrotic cells and their released components are involved in the neutrophilic EMR2 increase in vitro. To destroy complement activity, FCS was inactivated, but it increased neutrophilic EMR2 as native FCS (Table 2).

To further examine the role of FHR1, we screened volunteers for the homozygous deletion of a chromosomal fragment containing the *CFHR1* gene (Δ*CFHR1*), which has a frequency between 4.9–25.0% in White European populations [41]. In cultures with 10% autologous serum, the increase in EMR2 at neutrophils was also seen in two volunteers with a homozygous *CFHR1* deletion (Figure S4a,b). Thus, in vitro FHR1 is not likely to be involved in neutrophilic EMR2 increase. Finally, we verified whether necrotic leukocytes and their released components additionally increased neutrophilic EMR2 compared to the control, but neither necrotic cells and their components alone nor combined with immobilized FHR1 obviously did so (Figure S4c).

### 3.8. EMR2 Increased on Neutrophils Also in Serum-Free Cultures

Surprisingly, serum withdrawal increased neutrophilic EMR2, as in the control with 10% FCS (Figure 5e,f). Leukocyte scatters and viability were little changed, as seen in flow cytometry. Neutrophilic CD16 decreased, a sign of programmed cell death induction, but the addition of the caspase inhibitor Z-VAD-FMK did not prevent the neutrophilic EMR2 increase. Notably, the induction of intrinsic and extrinsic apoptosis by staurosporin and TNFα/cycloheximide, respectively, stopped the neutrophilic EMR2 increase (Figure 5e,f). Furthermore, CD16 disappeared and many leukocytes died, as seen in the scatters in flow cytometry.

In summary, stimuli increasing neutrophilic EMR2 were already present in and/or accumulate during culture. Notably, the lysis of red blood cells to isolate leukocytes generates cell debris, acting as DAMPs, but any other isolation method or enrichment of neutrophils without lysis also resulted in the generation of damaged/dying cells and cell debris easily seen via unbiased scatter analysis in flow cytometry. Thus, we are unable to exclude DAMPs in our in vitro experiments.

## 4. Discussion

Trauma is a ‘sterile’ inflammatory process in which neutrophils immediately recognize and are activated by endogenous host-derived DAMPs released by damaged/dying or necrotic cells [5,23]. The life cycle of neutrophils matches this function. Produced in reserve in the bone marrow, and partly attached to the endothelium in vessels, known as marginal pool, neutrophils are released after trauma [5]. They circulate only for a few hours before migrating into peripheral tissues where they fulfill their function in a short time frame. Overall, DAMP-induced inflammation is important for tissue repair and regeneration (reviewed in [42]).

Here, we recognized that EMR2 expression on circulating neutrophils shows a characteristic and uniform posttraumatic course in all injured patients. After a lag phase of several hours, the percentage of EMR2^+^ neutrophils and the EMR2 expression level on neutrophils increased and peaked two days after injury. Afterwards both parameters declined but frequently remained at a higher level compared to uninjured volunteers. This posttraumatic course of EMR2 appears to be independent of injury severity in patients with an ISS ≥ 9 and independent of the type of injury. However, very severely injured patients compared to severely/moderately injured ones have elevated absolute numbers of neutrophils; thus, more circulating neutrophils with advanced EMR2 levels are available in very severely injured patients. Consistent with our data, the increased expression of EMR2 on circulating granulocytes was seen in patients with non-infectious systemic inflammatory response syndrome (SIRS) compared to control subjects (*n* = 14/group) [14]. Furthermore, to evaluate biomarkers differentiating patients with SIRS from those with sepsis, neutrophilic EMR2 and CD11c were quantified once-only within 72 h of ICU admission in 103 patients, 83 of whom have sepsis, and in 50 healthy normal subjects [19]. Patients with SIRS had an increased prevalence of neutrophils expressing CD11c and EMR2. Sepsis increased additionally the percentage of CD11c^+^ but not of EMR2^+^ neutrophils, likely EMR2 is not involved in PAMP recognition.

Interestingly, in trauma patients and uninjured volunteers with a higher percentage of EMR2^+^ neutrophils, the circulating EMR2^−^ and EMR2^+^ neutrophils differed: the expression of CD62L was lower and that of CD11c was higher in EMR2^+^ cells, indicating that they were more activated or primed for activation. Myeloid cells get with EMR2 a receptor, which, after its activation, switches on pro-inflammatory cascades. The ligation of EMR2 by mAbs enhanced the inflammatory responses of neutrophils to a panel of stimuli [14] and modulated the production of multiple cytokines and the survival of LPS-stimulated neutrophils [15]. In THP-1 monocytic cells mAb-triggered EMR2 stimulation induces macrophage differentiation and inflammatory responses via the Gα16/Akt/MAPK/NF-κB signaling pathway [43]. Notably, EMR2 in blood-derived monocytes binds a complex consisting of necrotic-type cells and FHR1, a dominant trigger of inflammation [16]. Via PLC, the binding of FHR1 to EMR2 induces the NLRP3 inflammasome independent of complement, leading to the subsequent secretion of IL-1β [16]. Furthermore, the EMR2 potentiates recruitment of neutrophils [14]: the mAb-ligation of EMR2 enhanced adhesion but not rolling of neutrophils on TNFα-stimulated human umbilical vein endothelial cells (HUVECs) under shear conditions.

Whether EMR2^+^ are actually more activated in the circulation compared to EMR2^−^ neutrophils remains an open question. It is likely that EMR2 is acquired to adhere neutrophils to injured surfaces and/or to leave circulation. EMR2 binds dermatan sulfate, also named chondroitin sulfate B (CS-B), a glycosaminoglycan highly present in the extracellular matrix or at cell surfaces where it is linked to core proteins. Decorin in the skin and cartilage or thrombomodulin [44] and endocan [45] in endothelia are such dermatan sulfate-containing proteoglycans. Glycosaminoglycans are released after trauma [46,47]. EMR2 binds dermatan sulfate with its 4th EGF-like domain, existing only in the longest isoforms of EMR2(EGF1-4) and of its homologue CD97(EGF1-5) [11,48]. Indeed, the affinity binding of EMR2- and CD97-expressing myeloid U937 cells can be achieved in surface-bound dermatan sulfate but not in grafted heparan sulfate (CS-A) in vitro [49].

What are the stimuli/conditions increasing EMR2 expression on neutrophils after injury? To answer this question we cultured leukocytes, isolated by red blood cell lysis only, up to 17 h. Unexpectedly, already in controls, culture in medium/10% serum, EMR2 expression increased on neutrophils. This increase depends on extracellular, but not intracellular Ca^2+^ as experiments with extra- and intracellular Ca^2+^ chelators showed. The cytokines IL-6, IL-8, or CCL2, already increased at 1–8 h in our trauma patients, did not further upregulate neutrophilic EMR2 compared to the controls. The fact that even in serum-free cultures neutrophilic EMR2 increased indicates that it is independent from growth factors, and that the stimuli/conditions responsible for this process are already present in, arise, and/or accumulate during prolonged culture in the medium. Importantly, the induction of intrinsic and extrinsic apoptosis by staurosporin and TNFα/cycloheximide, respectively, stops the neutrophilic EMR2 increase in vitro.

Finally, the explanation that cell debris/particles, damaged/dying or necrotic cells, and/or released components, i.e. DAMPs, increase neutrophilic EMR2 during culture, and probably also in vivo, is logically consistent. It is impossible to culture isolated leukocytes or purified neutrophils without cell debris and/or damaged/dying or necrotic cells inside, as seen through unbiased flow cytometric scatter analyses of the freshly prepared cells and after 17 h of culture. After several efforts to purify neutrophils, with the highest gain being ~70%, we realized that we could not avoid cell damage and debris through purification steps. Instead the neutrophils were permanently impaired, they die faster in vitro. Our hypothesis that neutrophilic EMR2 increased through DAMPs is not contradicted by the lack of additional EMR2 upregulation when adding necrotic cells and their released components to the cultures because they are always present. We excluded FHR1 being involved in the increase in neutrophilic EMR2. Only when complexed with necrotic-type cells does FHR1 bind via EMR2 to primary monocytes in vitro [16]. However, two of our uninjured volunteers, whose neutrophils showed an EMR2 increase during culture, were *CFHR1*-deficient. Interestingly, one of the aGPCRs, BAI1/*ADGRB1*, binds apoptotic cells [50,51]. This is based on the binding capacity of the BAI1 N-terminal thrombospondin repeats for ‘eat-me’ signals in apoptotic cells and on the ability of the BAI1 C-terminal tail to facilitate cytoskeletal rearrangements. However, BAI1 is hardly expressed in myeloid cells [52].

Notably, we are unable to mimic in vitro the dynamic in vivo processes occurring in bone marrow, peripheral blood, and injured tissue in neutrophils after trauma when they are rapidly mobilized from all storage pools. The dramatic rise in circulating mature and immature neutrophil numbers turns out to be more obvious in very severely injured patients compared to moderately/severely injured ones, as confirmed in our research. Importantly, neutrophils circulate only for a few hours before migrating into injured or inflamed tissues. However, in our in vitro experiments, we investigated volunteers’ neutrophils, which are mainly of a resting phenotype [53]. The continuous replacement of circulating neutrophils by the storage pool-released cells, present in vivo, did not take place in vitro.

Neutrophil phenotypic heterogeneity is obviously altered following traumatic injury and is likely to contribute to the development of secondary complications [5,23,53,54]. In particular, the functional characterization of neutrophil subsets differently expressing surface molecules, such as CD16 and CD62L (plus CD11b, CD11c, CXCR2, and several others), received attention. After isolated blunt chest injury, CD62L and CXCR2 decrease compared to controls, a sign of neutrophil activation [55]. A CD16^bright(or high)^ CD62L^dim(or low)^ neutrophil subset appears during inflammation and is seen also after trauma [54]. It suppresses T cell proliferation. Furthermore, CD16^dim^ CD62L^bright^ neutrophils occur [54], which are banded, immature neutrophils, as seen via May–Grunwald staining. Importantly, neither the EMR2^−^ nor the EMR2^+^ neutrophils correspond to any of these subsets. We analyzed the CD16^high^ mature and the CD16^low^ immature neutrophil subsets separately for EMR2. In both subsets EMR2^−^ and EMR2^+^ cells are present and the EMR2 expression level and the percentage of EMR2^+^ cells similarly increased in both at 24–48 h after trauma.

Very recently, in injured patients and matched controls, eleven distinct neutrophil populations were resolved using mass spectrometry-based cytometry based on differential protein surface marker expression [53]. Trauma markedly altered the basal heterogeneity of neutrophil subsets seen in controls, with the loss of the dominant population of resting neutrophils and the expansion of two alternative neutrophil populations [53]. Thus, the increased expression of EMR2 on circulating neutrophils in our patients could also be the result of altered neutrophil subsets, differently expressing EMR2 after trauma.

## 5. Conclusions

After trauma, EMR2 expression on circulating neutrophils follows a characteristic and uniform posttraumatic course in all patients when a certain threshold of injury severity is reached. It is likely that EMR2 is a biomarker for injury in general; this hypothesis could be verified in patients who underwent a planned surgical intervention. Furthermore, EMR2 could be a biomarker related to a patient’s outcome late after injury, as the correlation analyses of EMR2 and patients clinical data 10 days after trauma suggest. Our in vitro data indicate that necrotic cells/DAMPs are likely to be the stimuli/condition upregulating EMR2 in vivo.

## Figures and Tables

**Table 1:** Patient characteristics according to the ISS.

	ISS < 25	ISS ≥ 25	*p*-Value *
patients (*n*)	9	25	
ISS	17 (10.5–18.0)	38 (30.5–44.0)	<0.001
age (years)	65.0 (39.0–74.0)	53.0 (32.5–68.0)	0.355
sex, female (*n*)	2	8	0.673
days at ICU (*n*)	2.0 (1.5–3.34)	11.0 (5.0–15.5)	<0.001
days in hospital (*n*)	15.0 (11.0–28.0)	19.0 (13.0–35.5)	0.335
death in hospital (*n*)	2	2	0.489
surgery between 1–8 h (*n*)	5	21	0.216
SOFA score ^#^ at 24 h	1.0 (0–3.0)	6.0 (2.0–10)	0.003

* Mann–Whitney U test; ^#^ Sequential Organ Failure Assessment (SOFA).

**Table 2:** Overview of conditions, stimuli, and substances tested to regulate EMR2 at neutrophils in vitro.

Serum/ Plasma (10%)	Stimulus/Condition	To Examine	Effect of Neutrophilic EMR2 #
FCS	-	control #	
human serum	uninjured volunteers, patients 24 h after trauma	stimuli present in patient’s sera	-
human plasma	uninjured volunteer, patient 24 h after trauma	stimuli present in patient’s plasma	inhibition compared to serum
FCS	1 mM EDTA	extracellular Ca^2+^ chelator	inhibition
FCS	10 μM BAPTA-AM	intracellular Ca^2+^ chelator	-
FCS	rh IL-1b, IL-6, IL-8, IL-17b; CCL2 (MCP1), TNFα	role of cytokines upregulated after trauma and/or inducing hepatic CRP	-
FCS	1 mM LPS	PAMP	-
FCS	force	mechanotransduction	-
inactivated FCS	-	complement	-
FCS/human serum	necrotic cells	DAMP	-
FCS/human serum	immobilized FHR1	FHR1	-
FCS/human serum	immobilized FHR1 + necrotic cells	FHR1 + DAMP	-
-	serum withdrawal	growth factors in serum	-
-	serum withdrawal + 20 mM Z-VAD-FMK	role of caspases	-
FCS	1 μM staurosporin	induction intrinsic apoptosis	no EMR2 increase, death neutrophils
FCS	5 ng/mL TNFα/ 10 µg/mL cycloheximide	induction extrinsic apoptosis	no EMR2 increase, death neutrophils

# leukocytes were cultured in RPMI/10% fetal calf serum (FCS); under these conditions, neutrophilic EMR2 increased.

## Data Availability

The data presented in this study are included in this published article or are available from the corresponding author upon reasonable request.

## Supplementary Materials

The following supporting information can be downloaded at: https://www.mdpi.com/2073-4409/12/22/2657/s1. Figure S1: Flow cytometric analysis of EMR2 expression on peripheral leukocytes; Figure S2: A 240 h time course of EMR2 expression on circulating neutrophils of uninjured volunteers; Figure S3: Posttraumatic time course of circulating cytokines; Figure S4: FHR1 is not involved in the increase in EMR2 expression on neutrophils in vitro; Table S1: Trauma classification; Table S2: Antibodies (Abs) and dyes used in flow cytometry.
