# Supplementary material for: The Posttraumatic Increase of the Adhesion GPCR EMR2/ADGRE2 on Circulating Neutrophils Is Not Related to Injury Severity
Source: Cells. 2023 Nov 20;12(22):2657. doi: 10.3390/cells12222657 (PMC10670733; doi:10.3390/cells12222657)
Supplement: Supplementary file 1 [file cells-12-02657-s001.zip › cells-2679046-supplementary.pdf]

cells

MDPI

Supplementary Materials

The Posttraumatic Increase of the Adhesion GPCR EMR2/ *ADGRE2* on Circulating Neutrophils Is Not Related to Injury Severity

Leyu Zheng [1], Moujie Rang [1], Carolin Fuchs[1], Annette Keß[1], Mandy Wunsch [1], Julia Hentschel [2], Cheng-Chih Hsiao [3] , Christian Kleber [1] , Georg Osterhoff [1#] and Gabriela Aust [1,4*#]

1 Research Laboratories and Department of Orthopaedics, Trauma and Plastic Surgery (OUP), Leipzig University and University Hospital Leipzig; Leipzig, Germany; leyu.zheng@medizin.uni-leipzig.de
2 Institute of Human Genetics, Leipzig University and University Hospital Leipzig; Leipzig, Germany ;
3 Department of Experimental Immunology, Amsterdam Institute for Infection and Immunity, Amsterdam University Medical Centers; Amsterdam, The Netherlands
4 Research Laboratories and Department of Visceral, Transplantation, Vascular and Thoracic Surgery (VTTG), Leipzig University and University Hospital Leipzig; Leipzig, Germany
* Correspondence: gabriela.aust@medizin.uni-leipzig.de
\# These authors contributed equally to this work

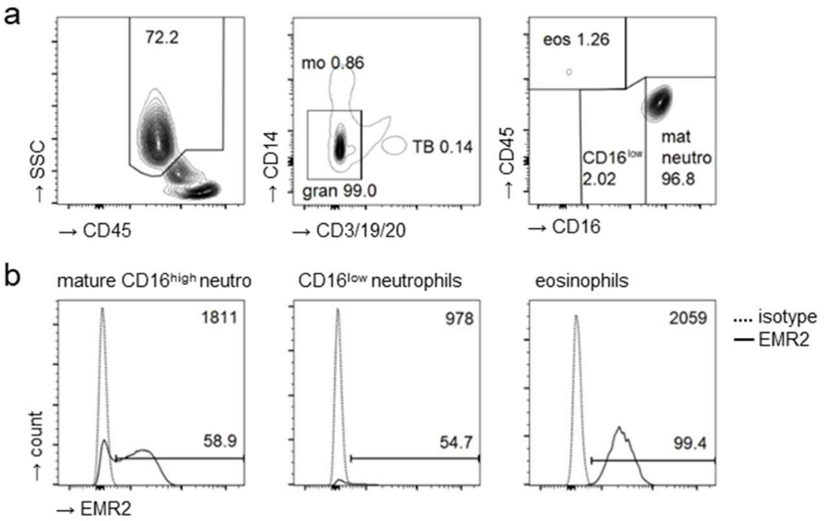

Figure S1. Flow cytometric analysis of EMR2 on peripheral leukocytes
a, b) Analysis of CD16^{high} and CD16^{low} neutrophils of an injured patient (ISS 32) 24 h after trauma. a) Main gating strategy for neutrophilic granulocytes (percentage of positive cells of all cells); mo monocytes, gran granulocytes, TB T- and B-cells, mat neutro mature neutrophils, eos eosinophils; b): Median fluorescence intensity (MFI; right upper corner) of isotype control- and EMR2 mAb-stained cells; the percentage of EMR2^{+} cells is indicated at the gate.

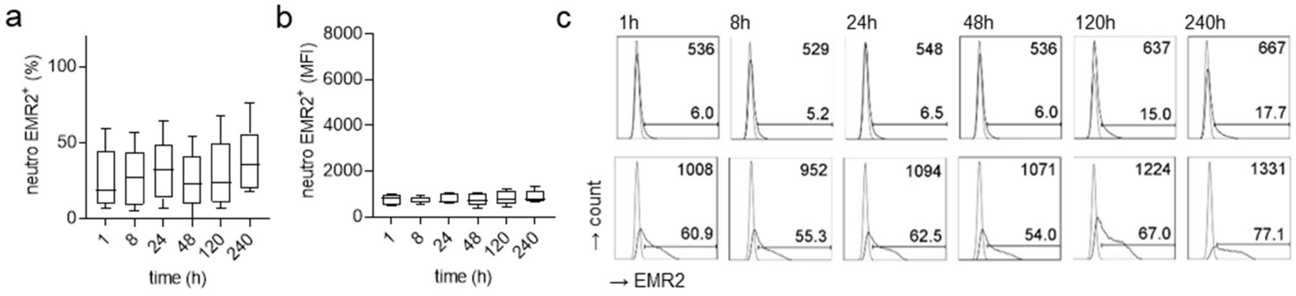

Figure S2. 240 h time course of EMR2 on circulating neutrophils of uninjured volunteers
(**a**) Percentage of EMR2⁺ neutrophils (a) and MFI of EMR2 on neutrophils (**b**), n= 5 volunteers.
(**c**) Time course of EMR2 on neutrophils in one typical volunteer (upper panel). In the lower panel a volunteer with a constantly high percentage of EMR2⁺ neutrophils is shown. The percentage of EMR2⁺ cells is indicated at the gate, MFI of EMR2⁺ cells is shown in the upper right corner.

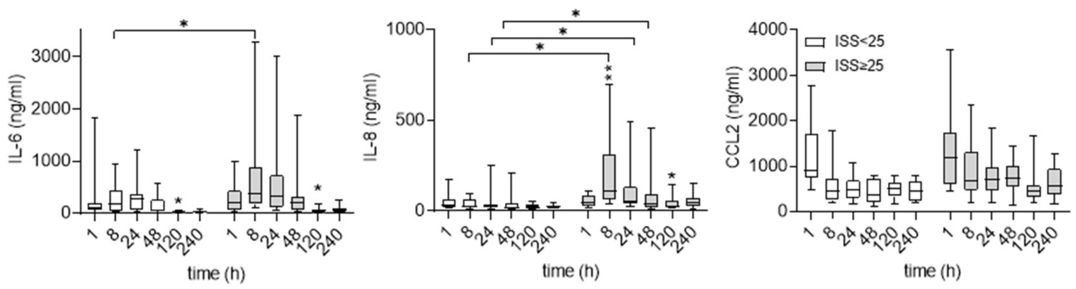

Figure S3. Posttraumatic time course of circulating cytokines
Comparison between patient groups ISS<25 (9-24) and ISS≥25, t-test (IL-6), U-test (IL-8, CCL2); comparison between consecutive time points in one patient group: ANOVA, only significant changes related to the previous time point are shown; *p<0.05).

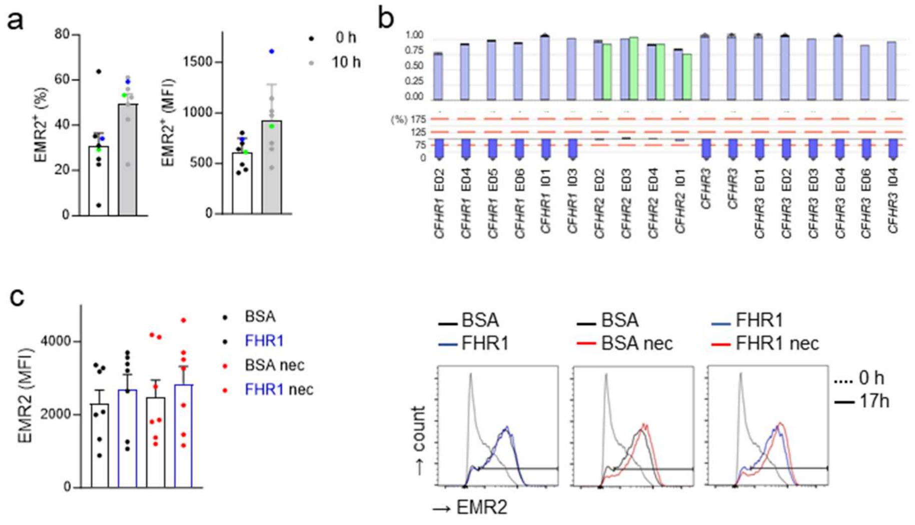

Figure. S4 FHR1 is not involved in the increase of EMR2 on neutrophils *in vitro*
(**a, b**) Leukocytes were cultured in RPMI1640/10% serum of the respective cell donor. EMR2 on neutrophils was quantified (**a**) before (0 h) and after 10 h by flow cytometry (n= 8 volunteers). 2 out of the 8 (2/8) volunteers, indicated by the colored dots in the graphs, were *CFHR1*-deficient, but they showed also a neutrophilic EMR2 increase; (**b**) MLPA analysis of the *CFHR1-3* target region. Upper trace: grey = mean value of 3 volunteers without, green = one volunteer with homozygous *CHFR1*/*CFHR3* deletion. Values between 0.75-1.25 display copy numbers of 2, as seen for the region *CFHR2*, while *CFHR1* and *CFHR3* revealed no signal for the target region, which means homozygous deletion. Lower trace: ratio between volunteers with/without deletion (%); 100 % means no difference between both. (**c**) Leukocytes were cultured either in BSA- or FHR1-coated wells with or without necrotic cells for 17 h. Afterwards the cells were stained and the expression of EMR2 on neutrophils was quantified by flow cytometry (n=7 donors). Right: One typical experiment of one donor is shown.

Table S1 Trauma classification based on the abbreviated injury scale (AIS)

Trauma group	Injuries	ISS<25 (n=9)	ISS≥25 (n=25)	p-value#
Neurotrauma	Traumatic brain injury	1	12	0.107
	Spinal cord injury	0	4	0.554
Facial trauma	Eye injury	1	3	1.000
	Fractured face	1	7	0.403
	Open wound face	3	1	0.048
Neck trauma	Esophageal trauma	0	1	1.000
	Tracheal trauma	0	1	1.000
Thoracic trauma	Internal organs injury	2	20	0.004
	Other injuries	4	17	0.254
Abdominal trauma	Internal organs injury	2	12	0.250
	Other injuries	0	6	0.162
Spinal trauma	Fracture	4	12	1.000
	Luxation/distortion	0	1	1.000
Upper extremities trauma	Fracture	4	11	1.000
	Luxation/distortion	1	1	0.465
Pelvic trauma	Fracture	2	16	0.052
Lower extremities trauma	Fracture	7	12	0.240
	Luxation/distortion	1	4	1.000

#Fisher's exact test

Table S2 Antibodies (Abs) and dyes used in flow cytometry

antigen, fluorophore	clone	company, catalog number
Abs for the clinical study		
CD14 BUV395	MφP9	Becton Dickinson GmbH (BD, Heidelberg, Germany) 563561
CD16 BUV737	3G8	BD, 612786
CD45 BB515	HI30	BD, 564585
EMR2 APC	2A1	Bio-Rad Lab GmbH (Feldkirchen, Germany), MCA2330A647T
CD3 BV510*	UCHT1	BD, 563109
CD19 BV510*	SJ25C1	BD, 562947
CD20 BV510*	2H7	BD, 563067
Abs and dyes additionally used for neutrophilic EMR2 analyses *in vitro*		
CD62L PE#	REA615	Miltenyi Biotec GmbH (Bergisch Gladbach, Germany) 130-114-151
CD11b Vio Bright B515#	REA713	Miltenyi, 130-131-818
CD11c PE-Cy7	B-Ly6	BD, 561356
EMR2 PE#	REA302	Miltenyi, 130-119-770
Fixable Viability Dye BV605		BD, 565694

*BV510 dump channel: CD3, CD19 and CD20 identify T and B cells, which are not of interest in this study; only $CD45^{+}$ $CD3/19/20^{-}$ cells are carried forward for analyses.

Disclaimer/Publisher's Note: The statements, opinions and data contained in all publications are solely those of the individual author(s) and contributor(s) and not of MDPI and/or the editor(s). MDPI and/or the editor(s) disclaim responsibility for any injury to people or property resulting from any ideas, methods, instructions or products referred to in the content.
